# *Mycobacterium tuberculosis* CarD, an essential global transcriptional regulator forms amyloid-like fibrils

**DOI:** 10.1038/s41598-018-28290-4

**Published:** 2018-07-04

**Authors:** Gundeep Kaur, Soni Kaundal, Srajan Kapoor, Jonathan M. Grimes, Juha T. Huiskonen, Krishan Gopal Thakur

**Affiliations:** 1 G. N. Ramachandran Protein Centre, Structural Biology Laboratory, Council of Scientific and Industrial Research-Institute of Microbial Technology (CSIR-IMTECH), Chandigarh, 160036, India; 2 Division of Structural Biology, Wellcome Trust Centre for Human Genetics, University of Oxford, Roosevelt Drive, Oxford, OX3 7BN, UK; 3 Diamond Light Source Ltd, Harwell Science & Innovation Campus, Didcot, OX11 0DE, United Kingdom; 4 Helsinki Institute of Life Science (HiLIFE), Biocenter 3, Viikinkaari 1, University of Helsinki, Helsinki, 00014, Finland

## Abstract

CarD is an essential global transcription regulator from *Mycobacterium tuberculosis (Mtb)* that binds RNA polymerase and activates transcription by stabilizing the transcription initiation complex. Available crystal structures have captured two distinct, monomeric and domain-swapped homodimeric, oligomeric states of CarD. However, the actual oligomeric state of CarD in solution and its biological relevance has remained unclear. Here, we confirm the presence of the homodimeric state of CarD in solution by using synchrotron-based small-angle X-ray scattering. Furthermore, by using biochemical and biophysical experiments, in addition to mass-spectrometry, transmission electron microscopy, and confocal imaging, we show that CarD is the first soluble cytosolic protein in *Mtb* which displays the tendency to form amyloid-like fibrils both *in vitro* as well as *in vivo*. We demonstrate that the deletion of the fourteen N-terminal residues involved in domain-swapping hampers amyloid formation, thus, suggesting that domain-swapping is crucial in amyloidogenesis. The discovery of the amyloidogenic property of an essential cytosolic global transcription regulator, CarD, in a pathogenic bacteria will further open up new frontiers in research.

## Introduction

Tuberculosis (TB), caused by *Mycobacterium tuberculosis (Mtb)*, was ranked among the top 10 causes of death worldwide for claiming 1.4 million lives worldwide in 2016^1^. A remarkable feature that makes *Mtb* one of the most successful pathogens is its ability to remain in a dormant/latent state for decades in the host before the onset of the disease as well as after the infection^2^. The formation of persister/dormant bacilli mainly involves fine tuning of transcriptional profile which involves coordination and interaction between the RNA Polymerase (RNAP) and the other transcription regulators and/or messenger molecules^3^. RNAP is an essential and key enzyme involved in transcription^4,5^. With the evolution in the biological complexity, RNAP have also evolved from the simplest single polypeptide chain in viruses to multi-subunit enzyme in bacteria, archaea and eukaryotes (Reviewed in^6^). Bacterial RNAP, a validated drug target^7^, is composed of five subunits (α_2_ββ’ω) which assemble to form a core-enzyme^4,5^. Transcription is a complex multistep process and several proteins like sigma (σ) factors, CarD, RbpA, DksA, Transcription-Repair Coupling Factor (TRCF) and many others, reversibly bind RNAP (core-enzyme) and aid transcription initiation, elongation or termination steps (Reviewed in^8,9^).

CarD, an essential global transcription regulator was discovered in 2009 and belongs to CarD_CdnL_TRCF family of transcription factors. CarD is a highly expressed protein having about 3000 molecules (~1.7 µM) per cell under exponential growth conditions^10,11^. The levels of CarD are highly up-regulated (~3 to 20-fold) in response to starvation, genotoxic, oxidative and antibiotic stress conditions and is required for persistence of *Mtb* within host^10^. The CarD depletion leads to upregulation of rRNA levels under stress conditions suggesting CarD regulates transcription of the rRNA operon^10^. However, recent studies demonstrate that CarD activates transcription by stabilizing an open promoter complex by preventing collapse of the transcription bubble^11–13^. Using fluorescent reporter based transcription assays Rammohan *et al*. proposed that CarD stabilizes open promoter complex by a two-tiered kinetic mechanism i.e. CarD activates transcription by both increasing the rate of opening and decreasing the rate of bubble collapse^11^. Weiss *et al*., reported that the interaction of CarD with RNAP β-subunit is responsible for mediating *Mtb* viability, rifampin resistance, and pathogenesis^14^. Hence, disrupting CarD/RNAP β-subunit interaction has been proposed as an alternate strategy for drug development^14^. Since *Mtb* transcription machinery is less stable than that of fast growing bacteria such as *E*. *coli*^12^, to partially compensate for low stability, CarD and RbpA, two essential transcription factors, act synergistically to activate transcription in *Mtb*^15–17^. Garner *et al*., 2017 demonstrated that affinity of CarD with RNAP is directly proportional to the growth rate in *Mtb* and is independent of 16S rRNA levels, thus indicating that growth rate and rRNA levels can be uncoupled in *Mtb*^16^.

**Figure 1:**
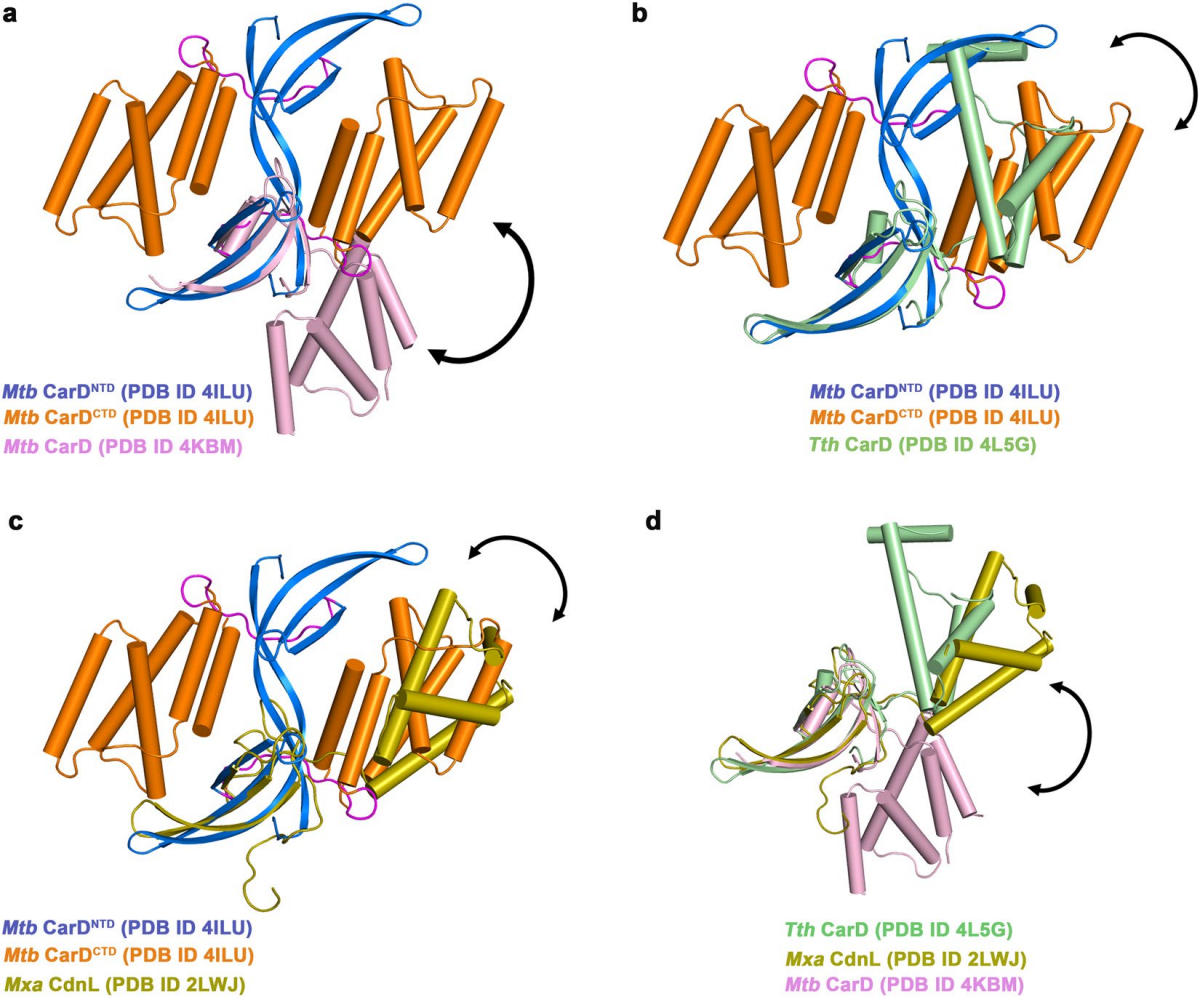
The comparative structural analysis of four full-length structures of CarD_CdnL_TRCF family of proteins. CarD consists of two domains, NTD (marine) and CTD (orange), linked with a flexible linker (magenta). The NTDs of the available structures of the CarD_CdnL_TRCF family of proteins (**a**) *Mtb* CarD (light pink; PDB ID 4KBM) (**b**) *Tth* CarD (light green; PDB ID 4L5G) (**c**) *Mxa* CdnL (olive; PDB ID 2LWJ) were superposed upon domain-swapped dimeric *Mtb* CarD (PDB ID 4ILU). (**d**) The crystal structure of *Tth* CarD and the NMR structure of *Mxa* CdnL were superposed using monomeric *Mtb* CarD (magenta; PDB ID 4KBM) as a reference. Structural comparison reveals different conformational states of CarD observed in available structures (indicated by arrows), thus, suggesting flexible nature of the CarD_CdnL_TRCF family of proteins.

*Mtb* CarD and its homologs, alone or in complex with RNAP, have been structurally characterized. Gulten *et al*., solved the structure of monomeric *Mtb* CarD in complex with RNAP β1-β2 domain of β subunit of RNAP and reported that CarD^CTD^ (C-terminal domain of CarD) binds DNA nonspecifically^18^. Kaur *et al*., solved the crystal structure of *Mtb* CarD alone and reported that CarD adopts a quasi-domain swapped dimeric architecture (Fig. 1a)^19^. The crystal structure of *Thermus thermophilus (Tth)* CarD (Fig. 1b)^20^ and NMR solution structure of *Myxococcus xanthus (Mxa)* CdnL^21^, homologs of *Mtb* CarD were also reported recently (Fig. 1c). Recently, Bae *et al*., reported the crystal structure of *Tth* transcription initiation complex with CarD and demonstrated the role of CarD in preventing bubble collapse and in stabilizing the transcription initiation complex^22^.

The available structural studies suggest that CarD can exist in two distinct oligomeric states, hence the current study was primarily aimed at resolving the oligomeric state of CarD in solution. Synchrotron-based small-angle X-ray scattering (SAXS) experiments suggest that CarD adopts homodimeric architecture in solution at higher μM range concentrations. We also show that CarD has a propensity to form SDS-resistant higher order oligomers in solution. Surprisingly, we discovered that CarD has the tendency to form amyloids both *in vitro* as well as *in vivo*. This makes CarD the second bacterial cytosolic transcription factor, after *Clostridium botulinum* Rho, a transcription termination factor^23^, and the first *Mtb* cytosolic protein that has been observed to form amyloid-like fibrils.

## Results

### Comparative analysis of the available structures of *Mtb* CarD and its homologs

Four full-length structures belonging to CarD_CdnL_TRCF family of transcription factors have been deposited in the Protein Data Bank (PDBIDs 4ILU, 4KBM, 4L5G and 2LWJ). Kaur *et al*., 2014 reported the crystal structure of domain-swapped homodimeric *Mtb* CarD (Fig. 1a), while, Gulten and Sacchettini reported that monomeric CarD binds RNAP β in 1:1 stoichiometry (Fig. 1a)^18,19^. *Tth* CarD has been crystallized as a monomer (Fig. 1b)^20^ and in-solution data for the oligomeric state is unavailable. In case of *Mxa* CdnL, NMR and analytical ultracentrifugation studies have indicated that monomers and dimers co-exist in solution (Fig. 1c)^21^. Furthermore, bacterial two hybrid and size-exclusion studies have suggested that N-terminal region of CdnL interacts with itself and forms homodimers in solution^21,24^. The solution structure of *Mxa* CdnL reveals high flexibility in the first six N-terminal residues^21^. Superposing the N-terminal domains of the *Mtb* CarD and its homologs, reveals differences in the conformational states of CarD, thus, highlighting the flexible nature of linker in the protein (Fig. 1d). Srivastava *et al*. reported that *Tth* CarD is rigidly maintained^20^ whereas our comparative analysis of all the full-length structures of CarD_CdnL family of proteins suggest that CarD may adopt functionally relevant multiple conformational states in solution.

### CarD exists as a homodimer in solution

**Figure 2:**
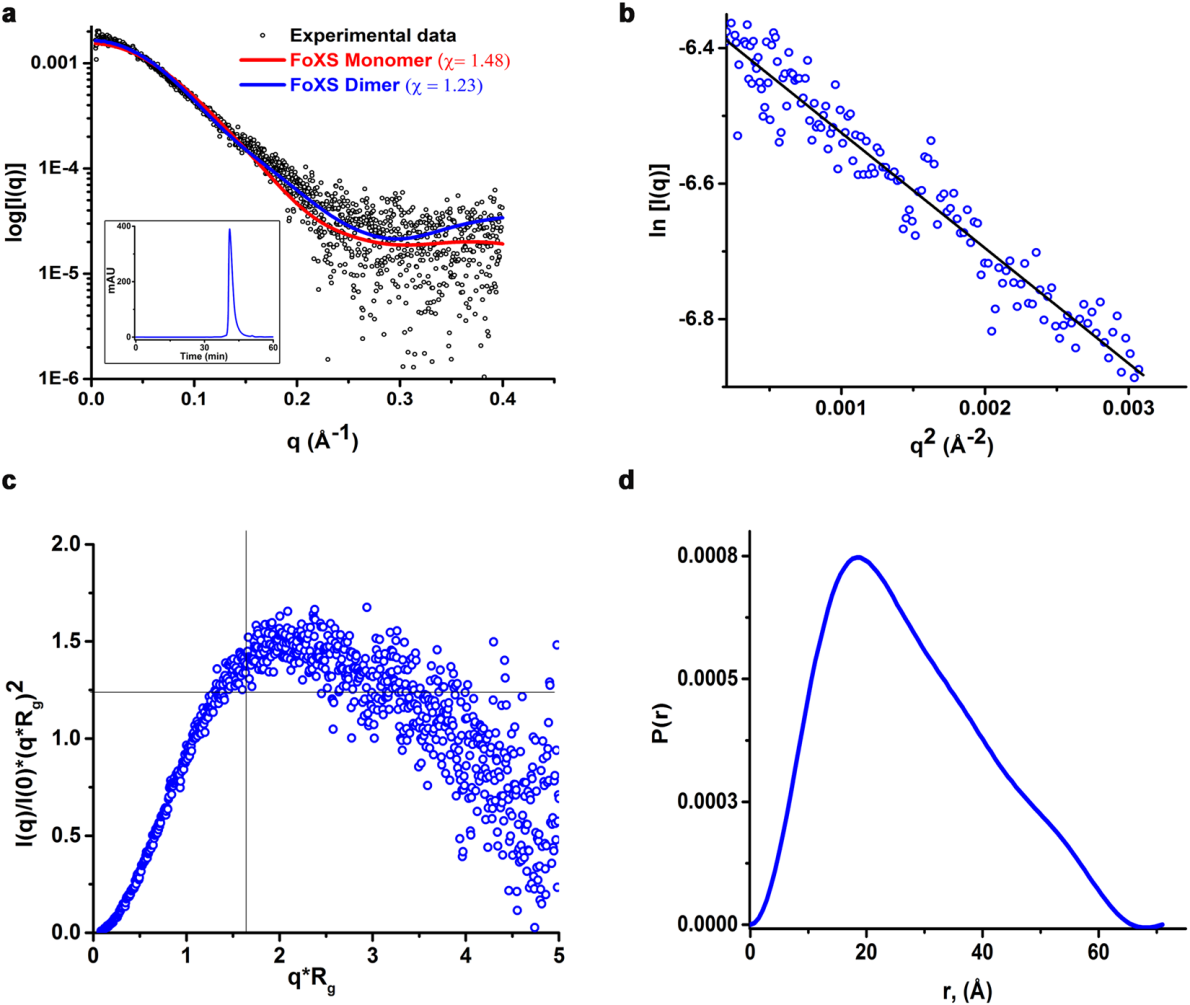
The SEC-SAXS analysis of CarD. (**a**) Scattering data obtained for CarD at 277 μM concentration. Inset shows the in-line SEC-trace of the CarD. CarD elutes as a single peak with no apparent signs of aggregation. FoXS web server analysis reveals that the theoretically calculated scattering profile from domain-swapped dimeric crystal structure (blue) matches well (χ = 1.23) with the experimentally observed scattering profile (black) whereas the monomeric CarD (red) shows relatively poor fit (χ = 1.48). (**b**) The linear low-q region (0.04–0.18) of the scattering curves were used for the Guinier analysis. (**c**) Kratky analysis of the CarD suggests CarD is well folded and flexible in solution. (**d**) Normalized pair distance distribution [P(r)] function suggests D_max_ of around 72 Å for CarD.

To further resolve the oligomeric state of CarD in solution, we performed synchrotron based size exclusion chromatography coupled with small angle X-ray scattering (SEC-SAXS) experiments. Since the crystal structure of the C-terminal his-tagged CarD (CarD^His^) was reported previously^19^, we used CarD^His^ for performing SEC-SAXS experiments and for performing all the other assays, we used both native and CarD^His^. CarD eluted as a single peak in the gel filtration chromatography having consistent values of radius of gyration (R_g_) across the peak (Fig. 2a inset, Supplementary Fig. 1a). The SAXS scattering intensity profile [I(Q)] as a function of Q, Guinier plot, Kratky Plot and normalized pair-distribution function [P(r)], are all shown in the Fig. 2(a–d). Analysis of the Guinier plots over a low q (between 0.004 and 0.18 Å^−1^), reveals that the purified protein was free of aggregation and inter-particle interference effects (Fig. 2b). Analysis of the Kratky plot and Porod-Debye plots^25^ suggests that CarD is well-folded globular protein (Fig. 2c). The Porod volume estimated from the SEC-SAXS data is 66,457 Å^3^. The estimated molecular weight (41 kDa) is in close agreement with the expected molecular weight of homodimeric CarD (38 kDa). The native-PAGE experiments were consistent with the SEC-SAXS results, suggesting the presence of dimeric CarD in solution (Supplementary Fig. 1b). In addition, we did not observe any monomeric species of CarD at three different concentrations (8, 30 and 80 μM) tested in the analytical size exclusion experiments (Supplementary Fig. 1c). The R_g_ obtained from Guinier analysis (R_g_ = 22.9 ± 0.064 Å) matches closely with the real space R_g_ derived from P(r) analysis (R_g_ = 21.6 ± 0.084 Å). The R_g_ and D_max_ (maximum dimension) calculated for the monomeric CarD (PDB ID 4KBM, coordinates extracted for CarD alone) were 17.6 ± 0.049 and 60 Å, respectively whereas the R_g_ and D_max_ calculated for dimeric CarD (PDB ID 4ILU) were 22.9 and 72 Å, respectively (Fig. 2d). The R_g_ and D_max_ values obtained from the SAXS analysis match well with the R_g_ and D_max_ values calculated for the dimeric CarD using FoXS server^26^ and CRYSOL^27^, respectively.

**Figure 3:**
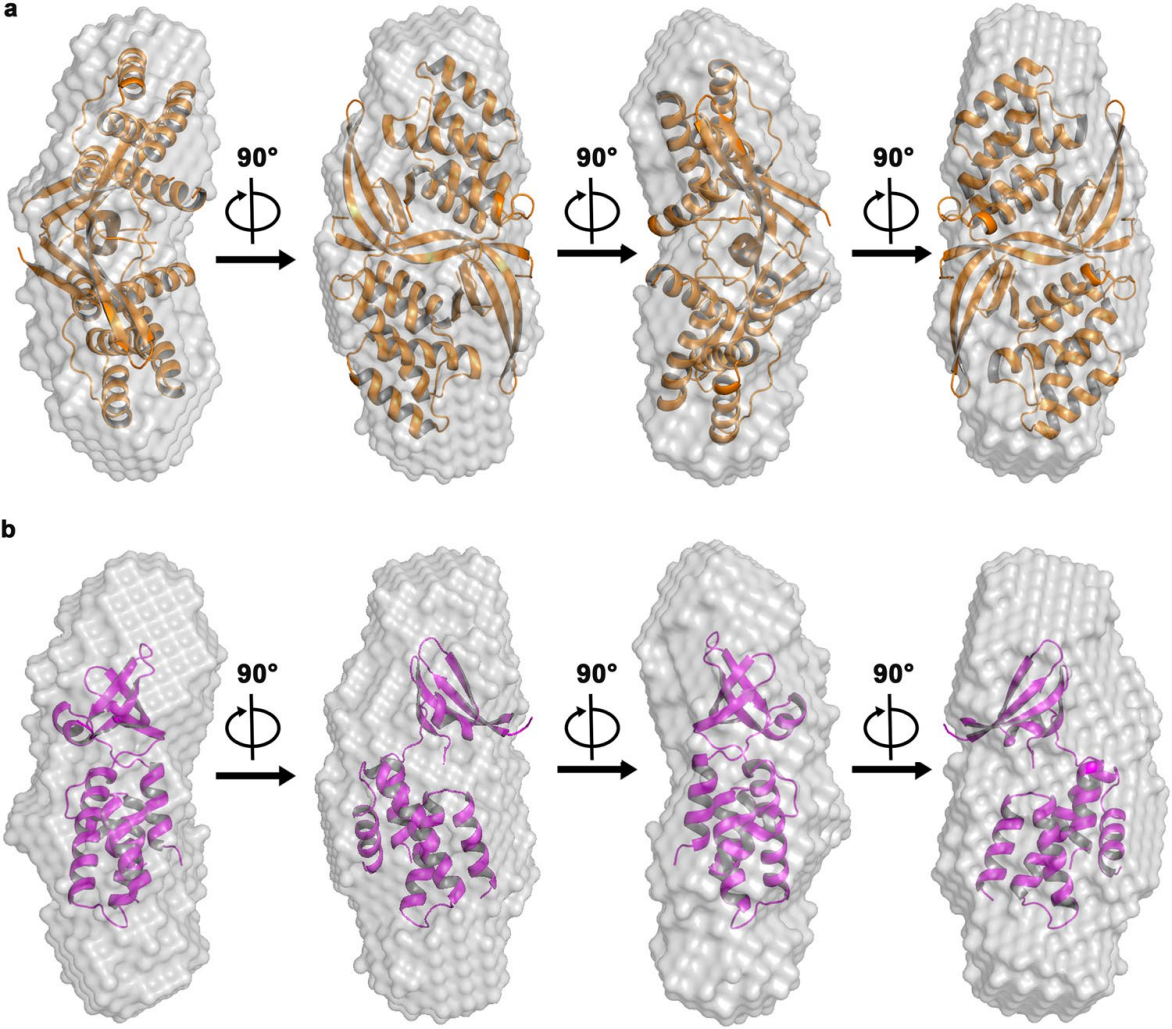
CarD exists as a homodimer in solution. SAXS-derived envelope of CarD was superposed using the available crystal structures of *Mtb* CarD using SUPCOMB. The crystal structure of the domain-swapped dimeric CarD fits well into the SAXS calculated envelope (NSD = 0.96, Fig. 3**a**) whereas the superposition of the monomeric CarD (NSD = 3.2, Fig. 3**b**) leaves multiple regions in the SAXS envelope unaccounted, suggesting a poor fit.

DAMMIF^28^ was used to generate 20 *ab initio* models which were superimposed, averaged and filtered by DAMAVER^29^ to generate the final model for envelope generation. We then superposed the available crystal structures of *Mtb* CarD (PDB ID 4ILU and 4KBM) into the SAXS derived envelope using SUPCOMB^30^ (Fig. 3). The domain-swapped dimeric crystal structure (Fig. 3a) superposed well whereas large unoccupied volumes were present when the monomeric CarD structure was superposed in the SAXS envelope (Fig. 3b). The NSD (normalized spatial discrepancy) values calculated using SUPCOMB for dimeric and monomeric CarD were 0.96 and 3.2, respectively, suggesting better fit for the dimeric CarD. Furthermore, the FoXS web server was used to compute the theoretical scattering profile from the crystal structures and compare it with the experimentally obtained scattering profile. The SAXS data acquired for CarD matches well with the theoretical scattering profile computed from the X-ray crystal structure of dimeric CarD (χ = 1.23) as compared to the monomeric CarD (χ = 1.48). Hence, our SAXS data further demonstrates the existence of homodimeric CarD in solution. Taken together these results show that CarD is monodisperse, homogeneous and exists as a homodimer in solution at the concentrations tested in our study.

### CarD undergoes reversible thermal folding in solution

**Figure 4:**
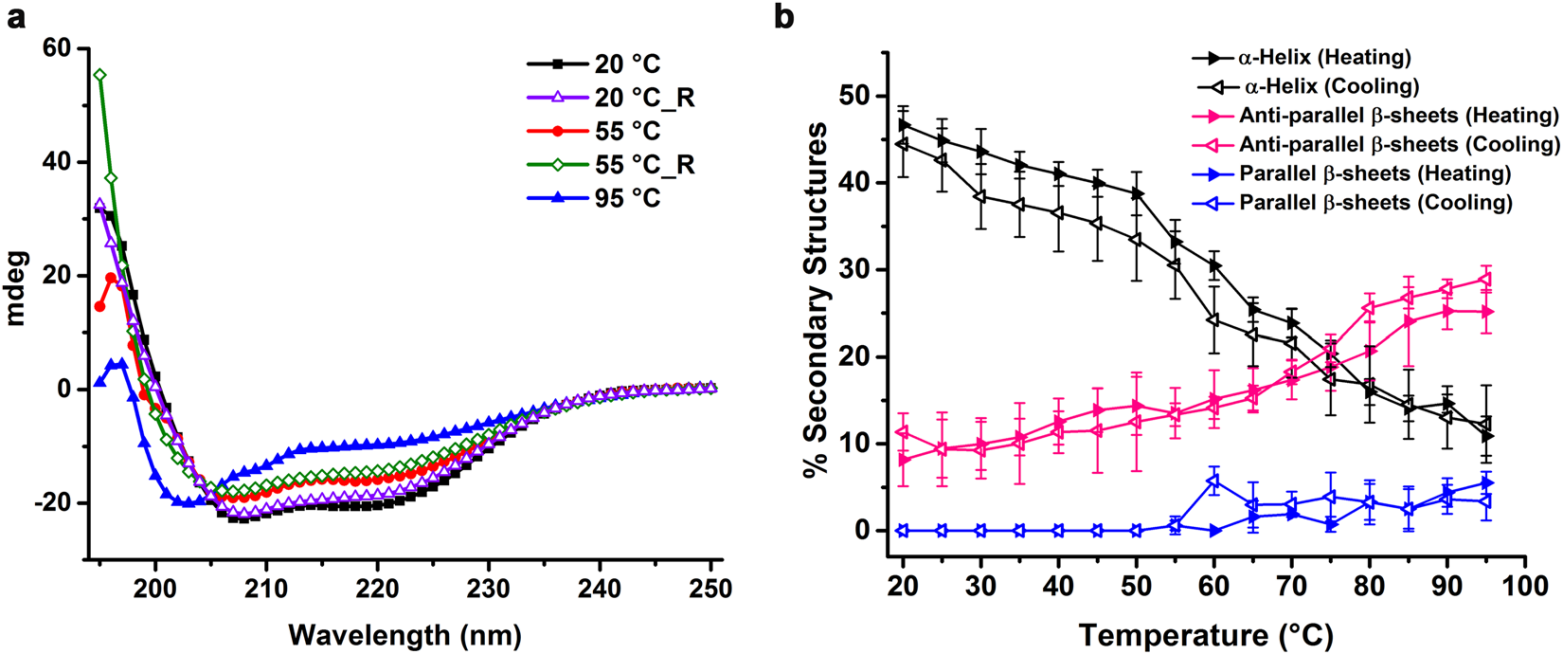
CarD undergoes reversible thermal folding in solution. (**a**) CD spectra obtained during thermal denaturation of CarD (10 μM). CarD was heated from 20 to 95 °C and then cooled from 95 to 20 °C. For clarity, only the representative CD spectra acquired at 20 °C, 55 °C and 95 °C during both heating and cooling (labeled with ‘_R’) have been plotted. The CD spectra reveal partial unfolding at elevated temperatures and refolding upon cooling with the presence of isobestic/isodichroic point at 205 nm. (**b**) The plot showing variations in secondary structural content of CarD with change in temperature. With the increase in temperature, the percentage of α-helices decreases and the percentage of anti-parallel β-sheets increases significantly.

Taken together, the crystal structure as well as the solution studies, both confirms the presence of domain-swapping and dimerization in CarD. We assume that such a domain-swapped dimer could form by partial unfolding of two monomers, followed by refolding and assembly into an intertwined form^31,32^. To test this hypothesis, we performed thermal denaturation experiments. Consistently, circular dichroism (CD) spectra revealed that CarD undergoes reversible thermal melting/folding with the presence of isosbestic point at 204 nm (Fig. 4a). The deconvolution of the CD spectra obtained as a function of temperature using the BeStSel program^33^, revealed a ~4-fold decrease in the α-helical content (both regular and distorted) when temperature was increased from 20 to 95 °C (Fig. 4b). On the other hand, there was a ~ 2.5-fold increase in the parallel and anti-parallel β-sheet content (primarily, right-handed twist angle increased by ~3-fold (Supplementary Fig. 2) with the increase in temperature. Interestingly, with the decrease in temperature (95 °C to 20 °C), the changes in the secondary structural contents showed the reverse trend (Fig. 4b). The presence of highly flexible six N-terminal residues as evident in the solution structure of *Mxa* CdnL^21^ and the ability to undergo reversible thermal denaturation strongly suggest that CarD may undergo domain-swapping by partial unfolding followed by re-assembly with its counterpart.

### CarD has a propensity to form higher order oligomers in solution

**Figure 5:**
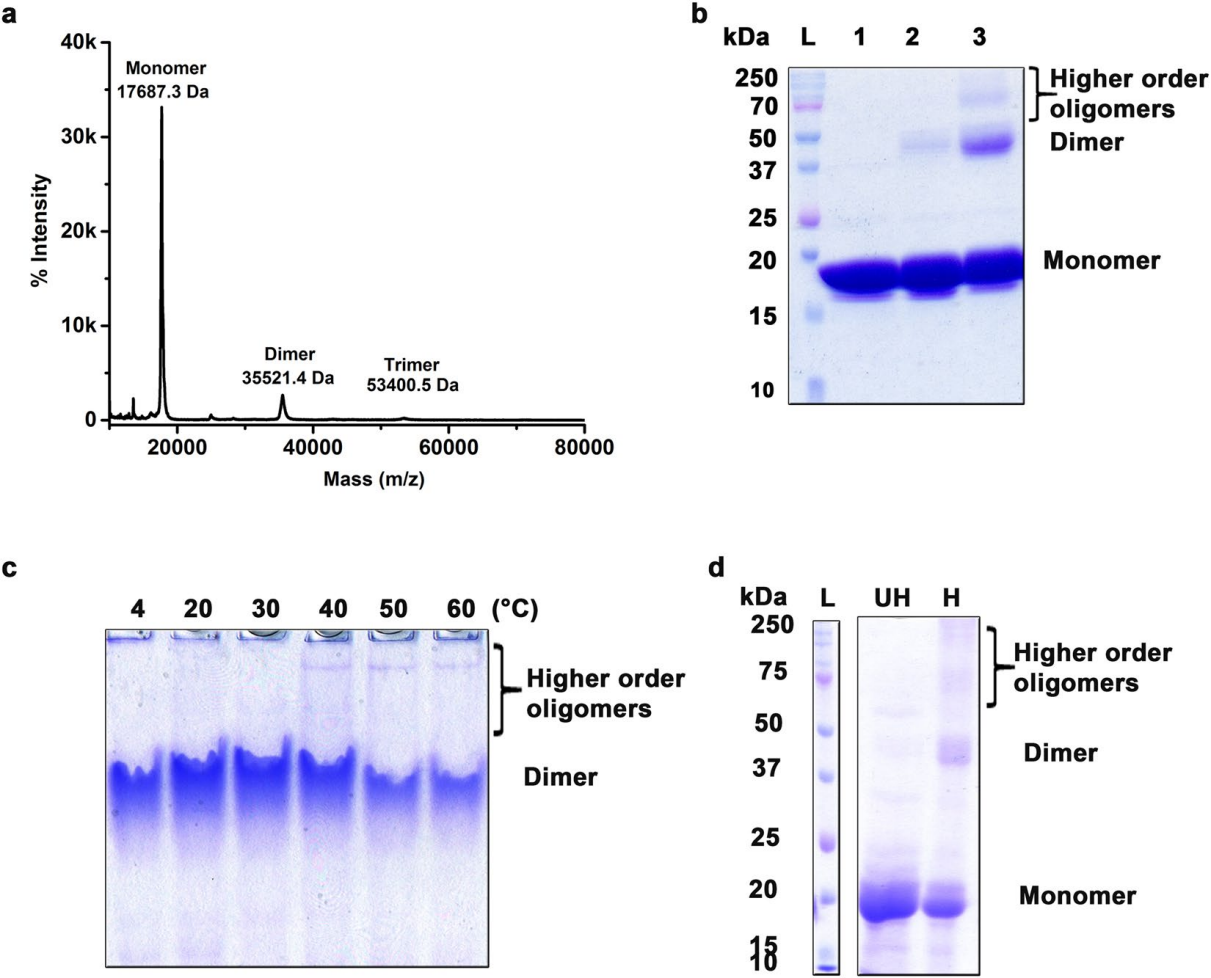
CarD has the tendency to form higher order oligomers in solution. (**a**) MALDI-TOF based intact mass spectrum analysis of CarD (50 μM) shows the presence of monomeric (17.687 kDa), dimeric (35.521 kDa) and trimeric (53.400 kDa) species. (**b**) Chemical cross-linking experiments performed using BS^3^ demonstrates that CarD (50 μM) has a tendency to form dimers, and higher order oligomers in solution. Lane 1, CarD in absence of BS^3^; lane 2, and 3, CarD in presence of 30-fold and 50-fold molar excess of BS^3^ respectively. L denotes the molecular weight ladder used as a standard. (**c**) CarD samples (50 μM) were incubated at 4, 20, 30, 40, 50, and 60 °C for 30 min and resolved in the native-PAGE (10%). Native-PAGE analysis suggests the formation of higher order oligomers with increase in temperature. (**d**) SDS-PAGE analysis of the thermally induced higher order oligomers of *Mtb* CarD (50 μM). L denotes the molecular weight ladder. UH and H represent the unheated and heated protein samples of CarD, indicating the formation of SDS-resistant dimers and higher order oligomers in the gel. The un-cropped images of the gels shown in **b–d** are provided in Supplementary Fig. 7a–c.

Domain-swapping in proteins has been implicated in the formation for higher order oligomers and even closed spherical shells^34–37^. We aimed to investigate whether domain-swapped CarD oligomers can further self-associate to form higher order oligomers in solution. We used mass spectrometry to determine the mass of native CarD. The MALDI-TOF linear mass spectrum of the intact CarD shows peaks corresponding to monomeric, dimeric, and trimeric species of CarD (Fig. 5a). In order to re-confirm our results, we also performed cross-linking experiments using BS^3^ [bis(sulfosuccinimidyl)suberate chemical cross linker. BS^3^ contains an amine-reactive *N*-hydroxysulfosuccinimide (NHS) ester at each end of an 8-carbon spacer arm. We incubated CarD with 30-fold and 50-fold molar excess of BS^3^ for 1 h at 4 °C and later quenched the reactions by addition of 50 mM Tris-HCl pH 8.0. Bands corresponding to the dimeric and higher oligomeric state species were observed on the SDS-PAGE (Fig. 5b). Taken together, these data suggest that CarD has a propensity not just to dimerize but also to form higher order oligomeric species in solution.

### CarD forms SDS-resistant higher order oligomers upon thermal denaturation

There is a strong correlation between the ability of a protein to form domain-swapped dimers and amyloids^38,39^. In several instances, domain-swapped oligomers serve as precursors for the formation of the amyloid fibrils, including a prion protein^40,41^, cystatinC^42^ and GB1^43^. To further investigate whether higher order oligomeric species of CarD form amyloid-like fibrils, we performed thermal denaturation experiments. With the increase in temperature, dimeric CarD further self-associates to form higher order oligomers as suggested by the native-PAGE analysis (Fig. 5c). CarD (50 μM) was incubated at 50 °C for 10 min and samples were analyzed on SDS-PAGE. Though the predominant population was monomeric, SDS-resistant dimers and higher order oligomers were formed at low efficiency as observed on the SDS-PAGE, a characteristic feature of the amyloids or pili proteins^44^ (Fig. 5d).

The formation of SDS-resistant higher-order oligomers led us to hypothesize that CarD may also form amyloids. We performed bioinformatics analysis using PASTA 2.0 web server to predict “Presence of Amyloid STructural Aggregation” in CarD^45^. The number of amyloid fibril regions predicted by PASTA is 2 and the best aggregation pairing energy is −5.406. According to Trovato *et al*.,^46^ PASTA based best aggregation pairing energy of < −4.0, indicates the presence of cross-β fibrillar aggregates in the structure. The aggregation pairing matrix predicts that Met1-Tyr11 N-terminal region of CarD is involved in the self-aggregation and pairing. Amyloid fibril regions are no longer detected in PASTA 2.0 server if we delete eleven N-terminal residues of CarD, suggesting that the region involved in domain-swapping may also be crucial for the formation of amyloid fibrils. We created a CarDtr construct (CarD^15–162^) and performed thermal denaturation experiments. Upon thermal denaturation, no visible precipitates were observed in CarDtr and no SDS-resistant higher order oligomers were detected on the SDS-PAGE, suggesting that N-terminal residues are crucial for the formation of SDS-resistant higher order oligomers (Supplementary Fig. 3).

### CarD forms amyloid-like fibrils in solution

Our results indicated that N-terminal region of CarD encompassing β1 is involved in both domain-swapping^19^ as well as in the formation of SDS-resistant higher order oligomers, so, we hypothesized that it may be crucial for the amyloid-fibril formation as well. So, we further investigated the ability of CarD to form amyloid-like fibrils using combination of ThioflavinT (ThT) fluorescence assay and transmission electron microscopy experiments.

**Figure 6:**
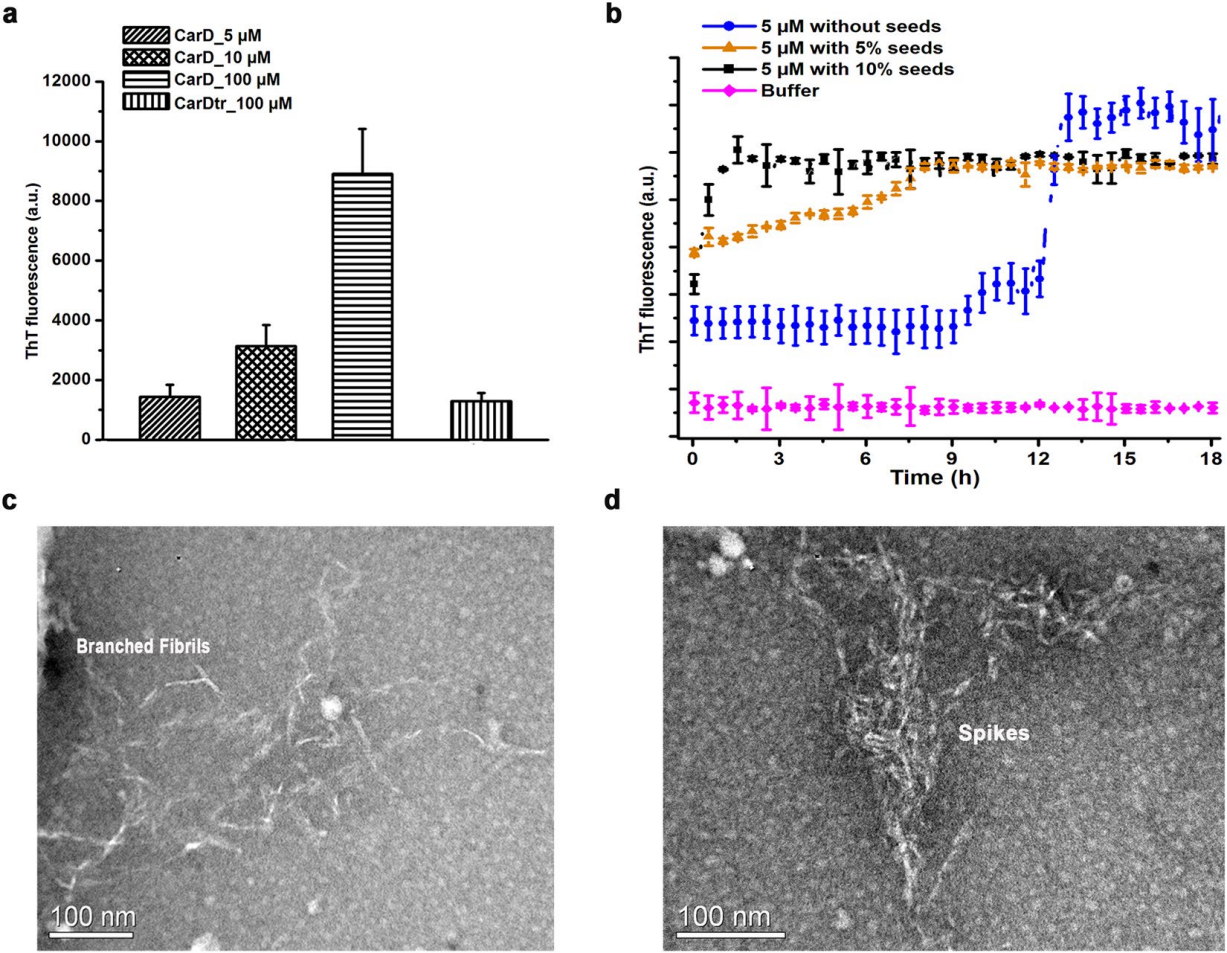
CarD forms amyloid-like fibrils in solution. (**a**) Thioflavin T fluorescence assay: Staining of native CarD (5, 10 and 100 μM) and CarDtr (100 µM) with amyloid specific fluorescent dye, Thioflavin T (ThT) at 37 °C after 15 min shows enhanced fluorescence intensity in case of native CarD as compared to CarDtr. The average fluorescence intensity and standard error bars have been calculated from three independent experiments. (**b**) The kinetics of amyloid-like fibril formation of native CarD (5 μM) in the absence and presence of 5% and 10% seeds monitored at 37 °C. The ThT fluorescence intensity was measured using excitation wavelength of 440 nm and emission wavelength of 482 nm. (**c** and **d**) The transmission electron microscopy (TEM) was used to visualize the CarD amyloids. The electron micrographs reveal several branched amyloid-like fibrils (**c**) and spikes (the aggregates of amyloid-like fibrils; **d**).

The presence of amyloid fibrils was detected by ThT dye based assay, a fluorescent probe commonly used to monitor the formation of amyloid fibrils *in vitro*^47^. Upon binding amyloid fibrils, ThT gives a strong fluorescence signal at approximately 482 nm when excited at 440 nm. We incubated varying concentrations of CarD (5, 10 and 100 μM) with ThT at 37 °C for 15 min and observed a significant increase in fluorescence intensity (Fig. 6a). On the other hand, when we incubated CarDtr (100 μM) with ThT, no significant increase in fluorescence intensity was observed (Fig. 6a), thus, indicating that the N-terminal region encompassing β1 is essential for amyloid formation. Further, the kinetics of formation of amyloid fibrils by CarD (5 µM) was monitored by ThT fluorescence at 37 °C for a period of 24 h (Fig. 6b). The fibrillation pattern resembles a characteristic sigmoidal curve consisting of a long lag (nucleation) phase, an elongation phase followed by a stationary phase. While the higher concentrations of CarD (100 μM) reached saturation within about 200 mins of incubation at 37 °C, the lower concentrations of CarD showed a comparatively longer lag phase. To further test amyloidogenic property and to reduce the lag phase, we performed the seeding experiments by adding preformed nuclei or seeds (5% or 10%) to the sample. As expected, addition of seeds reduced the lag phase significantly (Fig. 6b) as compared to the unseeded CarD.

The formation of CarD amyloid-like fibrils was further confirmed by transmission electron microscopy (TEM). Electron micrographs reveal the presence of several branched fibrils (protofibrils), ~40–50 Å in diameter when imaged after 10 h of incubation (Fig. 6c). The aggregated amyloid fibrils, known as “spikes” were also visible in electron micrographs (Fig. 6d). However, we did not observe any fibrils for CarDtr sample in EM studies under identical conditions.

### CarD forms amyloid inclusions *in vivo*

**Figure 7:**
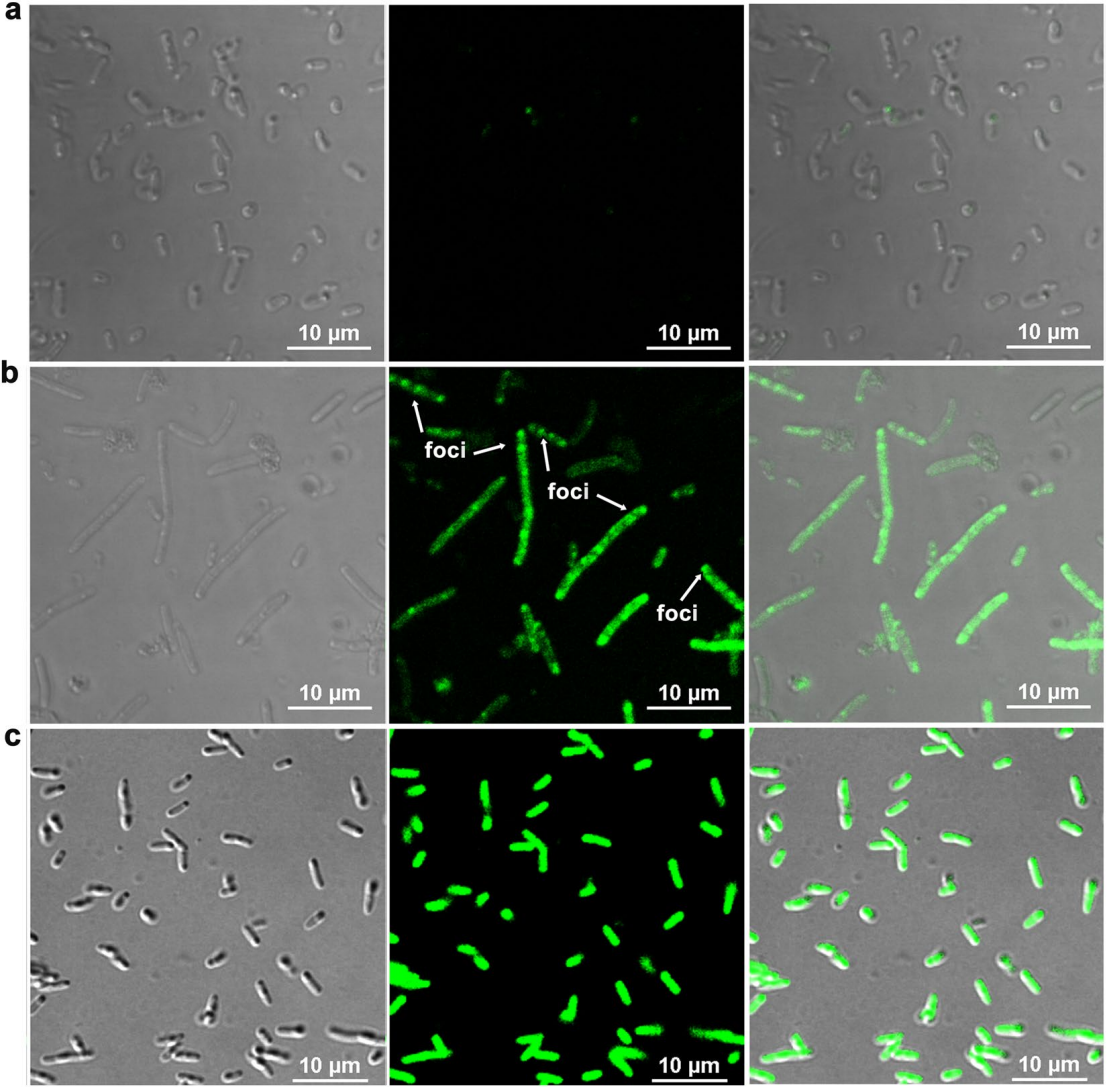
CarD forms amyloid inclusions *in vivo*. The confocal fluorescence microscopic images of bacterial cells over-expressing (**a**) empty vector, (**b**) CarD, and (**c**) CarDtr. The left, middle and right panels correspond to the phase contrast microscopy, fluorescence microscopy and merged images captured under UV light. Scale bar corresponds to 10 μm.

ThioflavinS (ThS), being cell permeable, is generally used to detect the presence of intracellular amyloid-like structures in live bacterial cells^48,49^. CarD is essential in Mycobacteria but absent in *E*. *coli*, hence, we used *E*. *coli* as an expression host for investigating *in vivo* amyloidogenic property of CarD. The bacterial (*E*. *coli)* cells carrying empty expression vector, over-expressing CarD or CarDtr were stained with ThS and monitored using confocal microscopy. *E*. *coli* cells expressing CarD exhibit strong green fluorescence along with the presence of several strong foci in majority of the cells thus, suggesting that CarD adopts amyloid-like conformations within the bacterial cells (Fig. 7b). In contrast, the *E*. *coli* cells carrying empty vector exhibit only residual background fluorescence (Fig. 7a). Furthermore, we could observe significant cell elongation in the bacterial cells expressing CarD. This observation correlates well with earlier studies which demonstrate that aggregation of the amyloidogenic proteins inside bacteria promotes severe cell division defects^50^. However, we did not observe cell elongation in cells over-expressing CarDtr compared to control cells. The *in vivo* confocal imaging of the *E*. *coli* cells over expressing CarDtr, stained with ThS, did not show strong fluorescent foci suggesting that CarDtr has a reduced ability to form intracellular amyloid inclusions (Fig. 7c).

## Discussion

**Figure 8:**
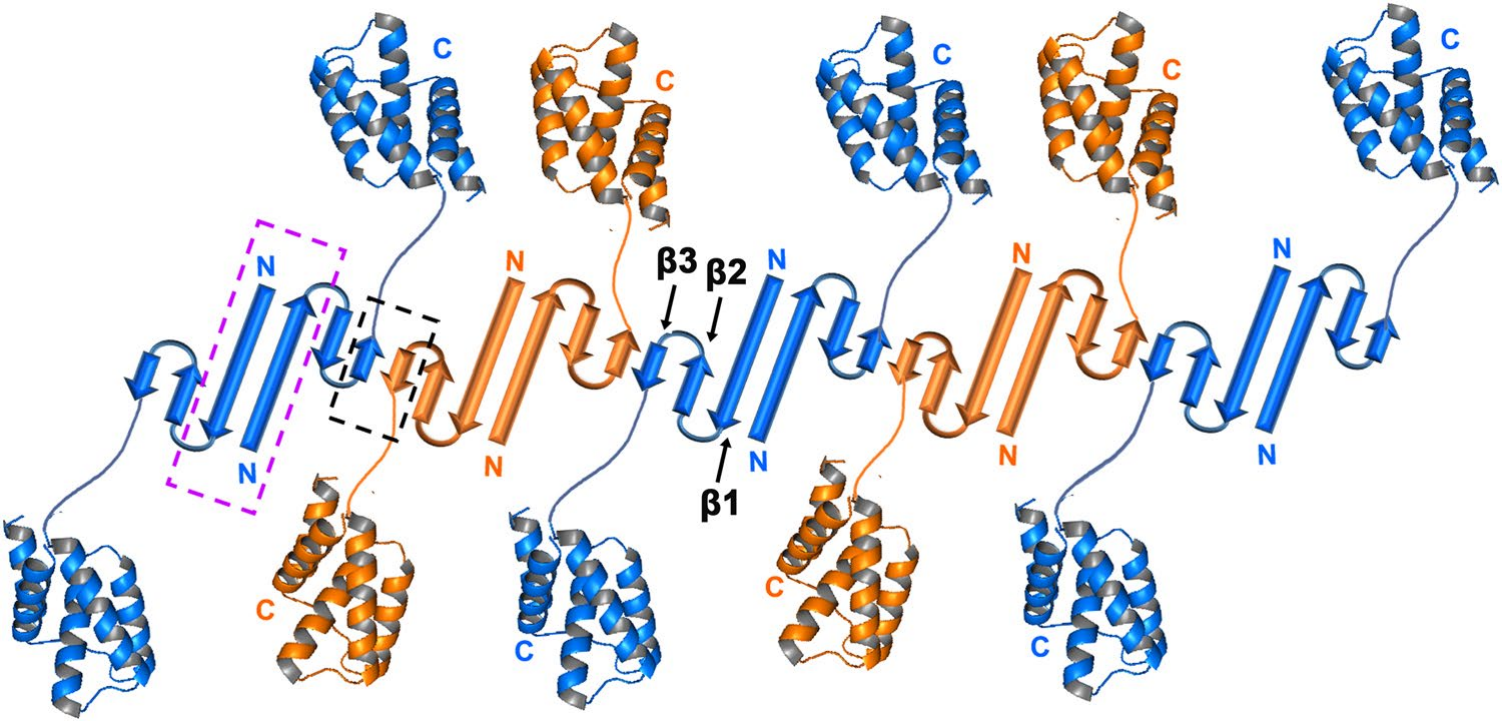
Schematic representation showing one of the probable mechanisms for amyloidogenesis in CarD. We assume that the N-terminal domains of CarD probably self-associate by β-sheet augmentation (shown in black box with broken line) leading to the formation of amyloid-like fibrils. The flexible linker may allow C-terminal domain to adopt conformation suitable for the formation of amyloids. The repeating homodimers of CarD are shown in blue and orange colors and the region responsible for domain-swapping is shown in magenta box with broken lines. For clarity, N-terminal domain has been shown as a topological diagram and C-terminal domain has been shown in cartoon representation.

In the current study, we attempted to resolve the oligomeric state of CarD in solution and therefore, determined its solution structure. Consistent with our previous findings^19^, we report that CarD exists predominantly as a dimer in solution at μM range of concentrations and has a tendency to form higher order oligomers. Concentration-dependent homodimerization has been previously reported in *Mxa* CarD^51^. However, *Mtb* CarD is the only example where the domain-swapped dimeric structure has been reported for the CarD_CdnL_TRCF family of transcriptional regulators^19^. The mechanisms or the factors which drive domain-swapping in CarD are currently unknown. One of the proposed mechanisms involved in domain-swapping is the ability of the proteins to partially unfold and re-fold in an inter-twined form along with their counter-part^32^. Our CD spectroscopy data suggests possible role of partial unfolding/refolding in mediating domain-swapping in CarD. We also report and demonstrate an interesting yet peculiar, amyloidogenic property of *Mtb* CarD, both *in vivo* as well as physiologically relevant conditions  in *in vitro* (5 μM). We observed that deleting the N-terminal fourteen residues involved in domain-swapping in CarD results in loss of self-association to form SDS-resistant higher order oligomers and hampers its ability to form amyloids in solution. Though in our solution studies we did not observe monomeric form of CarD under tested concentrations, Asmat *et al*.,^52^ have reported a small population of monomeric CarD in gel filtration experiments. The crystal structure of 1:1 stoichiometric complex of *Mtb* RNAP β1β2/CarD complex suggests that CarD exists as monomer as well in solution^18^. The protein sample in this study was prepared using co-expression and co-purification strategy suggesting probably in the presence of the binding partner, CarD exist as a monomer. CarD and RNAP population is similar in cells under normal growth conditions^11^. However, the levels of CarD transcripts increase from 5- to 20-fold under varying stress conditions^10^. So, under stress conditions CarD levels may probably reach in higher μM concentrations (>5 μM) which is in excess of its binding partner RNAP and hence, may self-associate to form homodimer. Further, self-association leads to the formation of higher order oligomers and amyloid-like fibrils in solution. Currently, it is not clear how and under what conditions, and if at all, monomer to dimer transition occurs in CarD. Based on our data, we propose a possible model for the formation of CarD amyloids where two N-terminal domains, having unpaired β strands probably self-associate by β-augmentation to form amyloid fibrils (Fig. 8). Interestingly, in the crystal structure of domain-swapped homodimeric *Mtb* CarD, this unpaired β3 was observed to interact with the flexible C-terminal His-tag and the latter also adopted a β strand conformation^19^. However, CarD^His^ and tagless CarD share similar functional properties suggesting there is no significant effect of histidine tag on the CarD function (Supplementary Fig. 4–6).

Usually amyloids are associated with the human diseases^53^ but there are several instances of functional bacterial amyloids formed by extracellular proteins such as Curli proteins from *E*. *coli*^54^, Type IV Pili from *Mtb*^44^, TasA from *Bacillus subtilis*^55^ and Microcin E492 from *Klebsiella pneumoniae*^56^. The presence of amyloidogenic properties in bacterial cytosolic proteins especially soluble transcription factors has been discovered recently^23,57,58^. *Cbo* transcriptional terminator Rho^23^, has been shown to form amyloids intracellularly in yeast and *E*. *coli* models. So, this field is still at its infancy and offers a wide opportunity to explore physiological relevance of amyloidogenesis in bacterial cytosolic proteins. Interestingly, while our manuscript was under communication, Takacs *et al*., analyzed the structures of all non-amyloid proteins present in PDB and predicted >500 soluble proteins with amyloid-like substructures, one of them being *Tth* CarD (PDBID: 4L5G) (https://arxiv.org/abs/1805.09758). Based on our results, we report that CarD is the first soluble cytosolic protein in *Mtb* which displays the tendency to form amyloid-like fibrils. As *Mtb* has a tendency to stay in a dormant/latent state for decades, hence there is a possibility of the formation of intracellular CarD amyloids over such a long period of incubation. So, it would be interesting to explore the physiological role, if any, of the amyloid-like fibrils formed by *Mtb* CarD. Future structural and functional studies are required to gain mechanistic insights into the phenomenon of domain-swapping mediated oligomerization and amyloidogenesis in CarD and similar behaving proteins.

## Materials and Methods

### Cloning, over-expression, and protein purification

The tagless construct of CarD was PCR amplified from *Mtb* genomic DNA and cloned into pYUB28b vector between NcoI and HindIII sites. The sequence of the clones was further confirmed by DNA sequencing. The positive clone of tagless CarD was transformed and expressed in *E*. *coli* strain BL21(DE3). The CarD overexpressing cells were grown in 1 L Luria Bertani (LB) media supplemented with Hygromycin (150 μg/mL) at 37 °C. When the cells reached OD_600_ = 0.5, the expression was induced by adding 0.3 mM IPTG and cells were further incubated for 16 h at 16 °C. The cells were harvested by centrifugation at 4 °C and the cell pellet was resuspended in 20 mM MES pH 6.0 and subjected to sonication. This was followed by centrifugation at 16,000 × g for 30 min at 4 °C. The supernatant was loaded immediately on DEAE-ion exchange chromatographic column pre-equilibrated with 20 mM MES pH 6.0. CarD eluted in the same buffer (20 mM MES pH 6.0) with the linear gradient from 0 to 1 M NaCl. The fractions corresponding to each peak were loaded on SDS-PAGE and the purified CarD fractions were pooled, concentrated and dialyzed in 20 mM HEPES pH 7.5 and 150 mM NaCl for further studies. The N-terminal deletion construct of CarD (CarD^15–162^, referred as CarDtr), was cloned in pNIC28-Bsa4 vector between NdeI and NotI sites. The His-tagged constructs of CarD (CarD^His^ and CarDtr) were purified as per the protocol in Kaur *et al*.^19^, except in this study, we used 20 mM HEPES pH 7.5 and 150 mM NaCl in all the purification steps. The identity of the protein samples were confirmed by MALDI-TOF mass spectrometry. The purity of all the protein samples were >95% as judged by the SDS- PAGE analysis. All the protein samples were further purified using gel filtration chromatography. The purified protein samples (500 μL each) were injected in Superdex S200 10/300 GL column pre-equilibrated with 20 mM HEPES pH 7.5 and 150 mM NaCl using AKTA purifier FPLC system (GE Healthcare) with a flow rate of 0.5 mL/min.

### Size-exclusion chromatography coupled with small angle X-ray scattering (SEC-SAXS)

SEC-SAXS was performed at the Diamond Light Source, Harwell, UK at B21 beamline. Inline SEC-SAXS data was collected using an Agilent HPLC equipped with Shodex column. Data were recorded on a Pilatus 2 M detector and purified samples of CarD (277 μM) were loaded onto the Shodex column pre-equilibrated in the running buffer of 20 mM HEPES pH 7.5 and 150 mM NaCl, at a flow rate of 0.018 mL/min at 4 °C. The primary reduction of the SAXS data and data processing were performed using ScÅtter (http://www.bioisis.net/ScÅtter) to obtain the radius of gyration (R_g_), the maximum particle dimension (D_max_), the excluded particle volume (Vp) and the pair distribution function [P(r)]. The molecular mass of the scattering particles was calculated using a method described by Rambo *et al*.^59^. Low resolution 3-D *ab initio* models were generated using DAMMIF software^28^ using the slow mode. Averaging the results of 20 independent runs using DAMAVER^29^ generated the dummy atom models which were superposed with the monomeric and domain-swapped dimeric crystal structures of *Mtb* CarD using SUPCOMB^30^. Structural visualization, analysis and figure preparation were performed using PyMoL (Schrödinger, LLC). FoXS web server^26^ was used to fit the experimentally derived scattering profile to the theoretically calculated scattering profile from the crystal structure. FoXS web server^26^ and CRYSOL^27^ were used for calculating R_g_ and D_max_ from the crystal structure and the experimental obtained SAXS data.

### CD spectroscopy and thermal melt assays

CarD or CarD^His^ (10 μM each) were dialyzed overnight into 10 mM sodium phosphate buffer (pH 7.5). CarD or CarD^His^ in a 1 mm path-length quartz cuvette were used to collect CD spectra in the 195–250 nm and 190–250 nm wavelength range, respectively, using JASCO J-810 spectrometer. Thermal melt experiments were performed from 20 to 95 °C and 95 to 20 °C temperature ranges with ramp rate of 1 °C/min. The spectra were recorded at every 5 °C with a scanning speed of 100 nm/min. The secondary structural content was deconvoluted using BeStSel Server^33^.

### Intact Mass Spectrometry analysis

Purified CarD (50 μM) or CarD^His^ or CarDtr were mixed with sinapinic acid matrix solution (50% Acetonitrile/50% water with 0.1% Trifluoroacetic acid) in 1:1 ratio and the mix was spotted on the MALDI plate. The spots were air dried and data were acquired in linear and positive ion mode keeping the laser intensity between 3000–3400 V (AB Sciex TOF/TOF 5800). The data were analyzed using Data Explorer software version 4.9 of Applied Biosystems.

### Chemical cross-linking

Cross-linking by BS^3^ [bis(sulfosuccinimidyl)suberate, Thermo Scientific, Catalog number 21580] was performed according to the method described in the user guide. CarD or CarD^His^ (50 μM each in 20 mM HEPES pH 7.5 and 150 mM NaCl) were mixed with 30-fold (1.2 mM) and 50-fold molar (1.5 mM) excess of BS^3^ and incubated on ice for 1 h. To terminate the cross-linking reaction, 50 mM Tris-HCl (stock solutions 1.5 M, pH 8.0) were added to the reaction mixtures after 1 h. The cross-linked proteins and the control protein samples were mixed with 2× SDS loading dye and resolved on the 15% SDS-PAGE.

### Native-PAGE analysis

Samples of purified CarD (50 μM) and CarD^His^ (50 to 716 μM) were incubated from 4 to 60 °C for 30 min. The heated samples were mixed with 2× non-denaturing gel loading dye (200 mM Tris-HCl, pH 6.8, 0.2% bromophenol blue and 20% glycerol). The continuous native-PAGE (10%) was cast and pre-run at 10 V/cm for 30 min at 4 °C. Electrophoresis was performed in 1× native-PAGE running buffer (25 mM Tris-HCl, pH 8.3, 192 mM glycine) according to the standard method at 4 °C for 2 h. The gels were stained using Coomassie brilliant blue followed by destaining using standard protocol.

### Thermal denaturation

To perform thermal denaturation experiments, CarD (50 μM), CarD^His^ (100 µM) or CarDtr (100 µM) were incubated at 50 °C for 10 min. The samples were then immediately mixed with 2× SDS loading dye and resolved on the 15% SDS-PAGE. The protein bands were visualized using standard staining and destaining protocols.

### Thioflavin T (ThT) fluorescence and fibrillation assays

Native tagless CarD (5, 10 and 100 μM), CarD^His ^(100 μM) and CarDtr (100 μM) were mixed with 10-fold molar excess of ThT and incubated for 15 min at 37 °C. Seeding experiments were performed where 5% and 10% seeds were transferred from the 100 μM CarD sample incubated at 37 °C for 5 h to 5 μM CarD. The ThT fluorescence intensity was measured using excitation wavelength of 440 nm and emission wavelength of 482 nm. The fluorescence measurements were recorded using Synergy H1 Hybrid multi-mode microplate reader. The kinetics for the formation of amyloid fibrils by CarD and CarD^His^ were recorded for a period of 24 h. The microplate reader was set at constant temperature of 37 °C and readings were acquired every 2 min with continuous orbital shaking between reads over a period of 24 h. The top of the each well was sealed using adhesive tape to minimize the rate of evaporation.

### Transmission Electron Microscopy (TEM)

Aliquots of 20 μL from samples containing CarD or CarDtr stained with ThT fluorescent dye were pipetted on a carbon-coated 300-mesh copper grid (Polysciences). Samples were diluted five-fold using 20 mM HEPES pH 7.5 and 150 mM NaCl buffer before loading on the grids. The excess sample was blotted and the grids were incubated with uranyl acetate solution (2%) for 2 min at room temperature followed by air drying. The grids were imaged using a JEM 2100 transmission electron microscope, operated at 200 keV (JEOL).

### Thioflavin S (ThS) staining of live bacterial cells

*E*. *coli* BL21(DE3) cells harboring CarD or CarDtr expression vector and empty expression vector control were grown at 37 °C in LB medium until optical density (OD_600_) reached 0.5. The protein expression was induced by addition of 0.3 mM IPTG and the cultures were further grown at 37 °C for 5 h after induction. Thereafter, 0.5 mL of each bacterial culture was centrifuged at 6000 × g, 25 °C for 2 min. The bacterial cultures were washed thrice with 1 × PBS, followed by their fixation using 4% paraformaldehyde at 37 °C for 30 min. The fixed bacterial cultures were washed thrice with 1 × PBS and further incubated with 125 μM ThioflavinS (ThS, Catalog number T1892, Sigma) at 37 °C for 30 min in the dark. The bacterial cultures were again resuspended and washed thrice with 1 × PBS. 10 μL of the sample was placed on top of the glass slide, covered with a coverslip and air-dried. The images were acquired using confocal fluorescence microscope (Nikon A1R), using a 100 × oil-immersion objective and 1 Airy unit aperture. The ThS stained samples were excited using an excitation wavelength of 405 nm and an emission wavelength of 482 nm.

## Electronic supplementary material

Supplementary Information
