# Supplementary material for: Mycobacterium tuberculosis CarD, an essential global transcriptional regulator forms amyloid-like fibrils
Source: Sci Rep. 2018 Jul 4;8:10124. doi: 10.1038/s41598-018-28290-4 (PMC6031611; doi:10.1038/s41598-018-28290-4)
Supplement: Supplementary file 1 — Supplementary Information [file 41598_2018_28290_MOESM1_ESM.pdf]

Supplementary information

Mycobacterium tuberculosis CarD, an essential global transcriptional regulator forms amyloid-like fibrils

Gundeep Kaur[1, 2], Soni Kaundal[1], Srajan Kapoor[1], Jonathan M. Grimes[2,3] Juha T. Huiskonen[2,4] and Krishan Gopal Thakur[1#]

[1] G. N. Ramachandran Protein Centre, Structural Biology Laboratory, Council of Scientific and Industrial Research-Institute of Microbial Technology (CSIR-IMTECH), Chandigarh-160036, India

[2] Welcome Trust Centre for Human Genetics, Division of Structural Biology, Oxford OX3 7BN, University of Oxford, UK

[3] Diamond Light Source Ltd, Harwell Science & Innovation Campus, Didcot, OX11 0DE, United Kingdom

[4] Helsinki Institute of Life Science (HiLIFE), Biocenter 3, Viikinkaari 1, University of Helsinki, 00014, Helsinki

[#] **Correspondence**

Krishan Gopal Thakur

Council of Scientific and Industrial Research-Institute of Microbial Technology, Chandigarh-160036, India.

E-mail: krishang@imtech.res.in

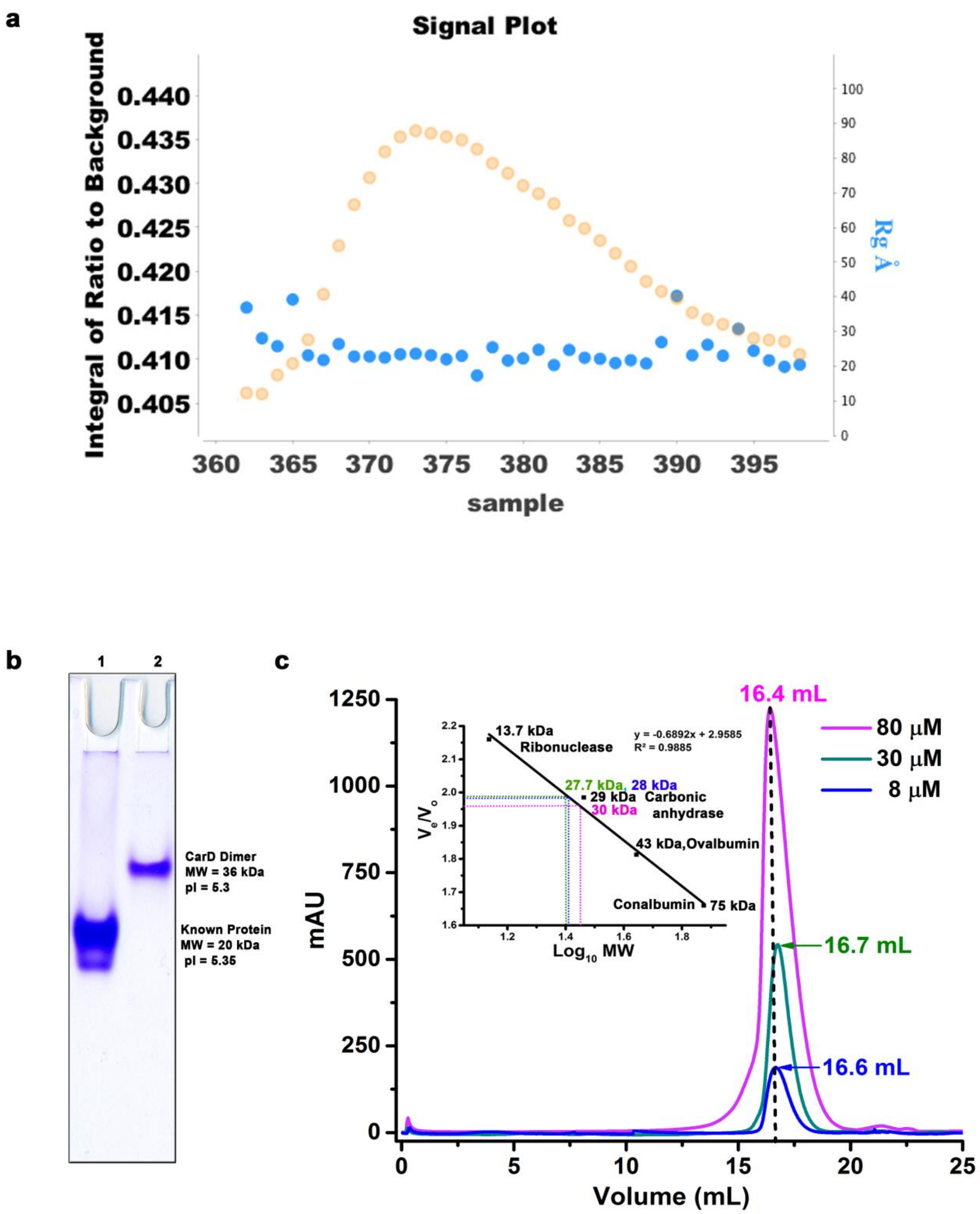

Supplementary Fig. 1: a) The determination of variation of R_g (in Å) across the SAXS I(0) peak. b) Native–PAGE analysis of CarD (pI: 5.3, Lane 2) along with a protein of known molecular weight (pI: 5.35, Lane 1) demonstrates that CarD exists as a dimer in solution. c) The analytical gel filtration profile of CarD at three different concentrations (8, 30 and 80 µM) monitored at 220 nm shows that CarD exists as a dimer in solution.

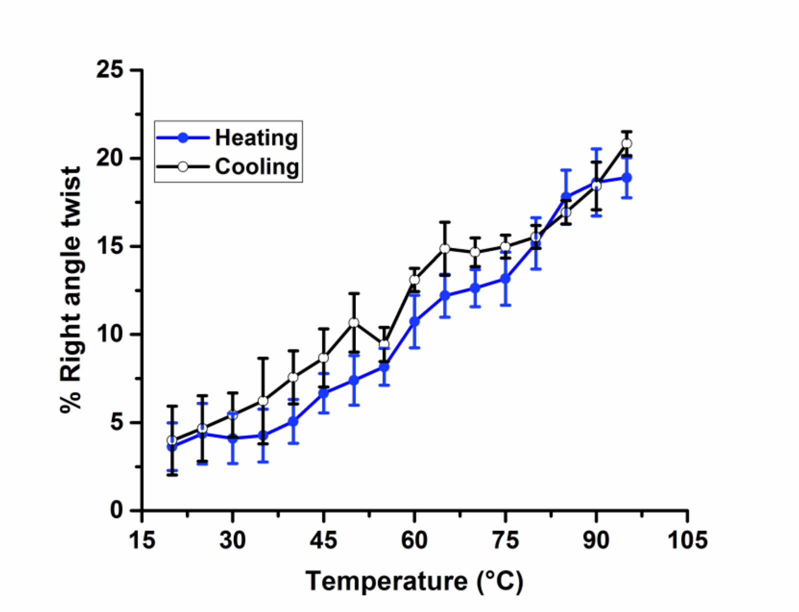

Supplementary Fig. 2: The plot showing variations in antiparallel right handed twist angle in CarD with change in temperature. With the change in temperature, the percentage of right handed twist angle changes by 7-fold.

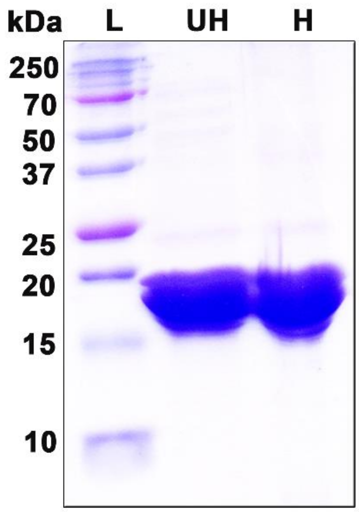

Supplementary Fig. 3: The SDS-PAGE analysis of the thermal denaturation of *Mtb* CarDtr. L denotes the molecular weight ladder. UH and H represent the unheated and heated protein samples of CarDtr, indicating the absence of higher order oligomers in the gel.

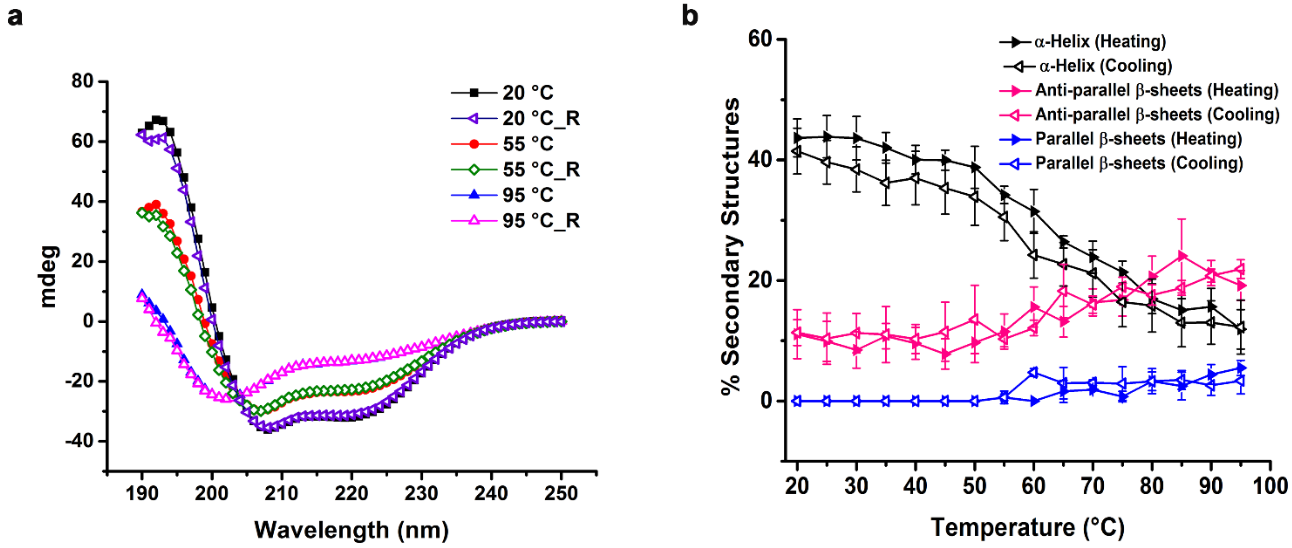

Supplementary Fig. 4: CarD^{His} undergoes reversible thermal folding in solution. (a) CD spectra obtained during thermal denaturation of CarD^{His}. CarD^{His} (10 µM) was heated from 20 to 95 °C and then cooled from 95 to 20 °C. For clarity, only the representative CD spectra acquired at 20 °C, 55 °C and 95 °C during both heating and cooling (labeled with '_R') have been plotted. The CD spectra reveal partial unfolding at elevated temperatures and refolding upon cooling with the presence of isobestic/isodichroic point at 204 nm. (b) The plot showing variations in secondary structural content of CarD^{His} with change in temperature. With the increase in temperature, the percentage of α-helices decreases and the percentage of anti-parallel β-sheets increases.

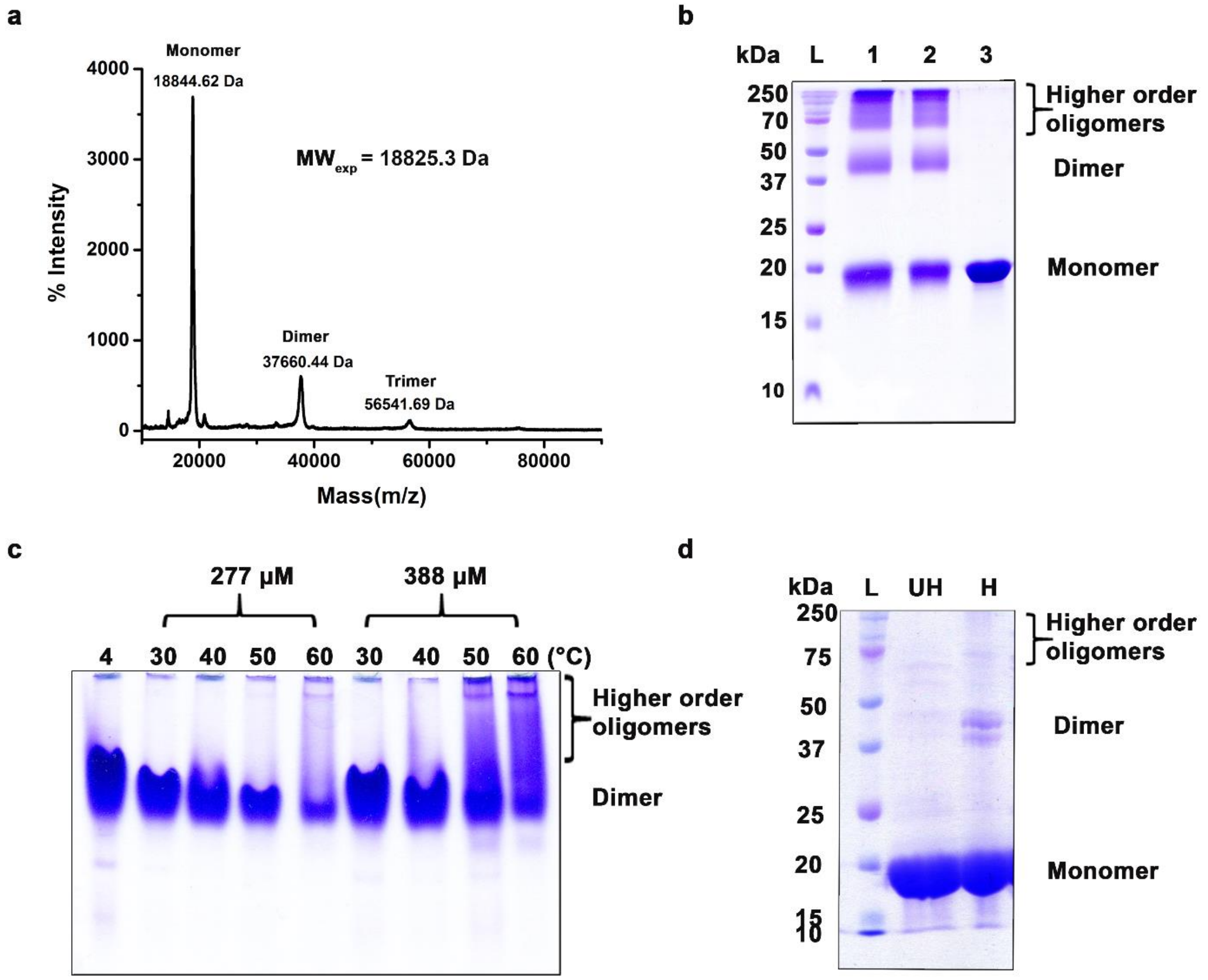

Supplementary Fig. 5: CarD^{His} has the tendency to form higher order oligomers in solution. (a) MALDI-TOF based intact mass spectrum analysis of CarD^{His} shows the presence of monomeric (18.84 kDa), dimeric (37.66 kDa) and trimeric (56.54kDa) species. The expected molecular weight of CarD^{His} is 18.82 kDa. (b) Chemical cross-linking experiments performed using BS^3 demonstrates that CarD has a tendency to form dimers, and higher order oligomers in solution. L denotes the molecular weight ladder used as a standard, Lane 1 and 2, CarD in presence of 30-fold and 50-fold molar excess of BS^{3}respectively, Lane 3, CarD^{His} in absence of BS^3. (c) CarD^{His} samples (277 µM and 388 µM) were incubated at 4, 30 °C, 40 °C, 50 °C, and 60 °C for 30 min and resolved in the 10% native-PAGE. Native-PAGE analysis suggests the formation of higher order oligomers with increase in temperature. (d) SDS-PAGE analysis of the thermally induced higher order oligomers of *Mtb*CarD. L denotes the molecular weight ladder. UH and H

represent the unheated and heated protein samples of CarD^{His}, indicating the formation of SDS-resistant dimers and higher order oligomers in the gel.

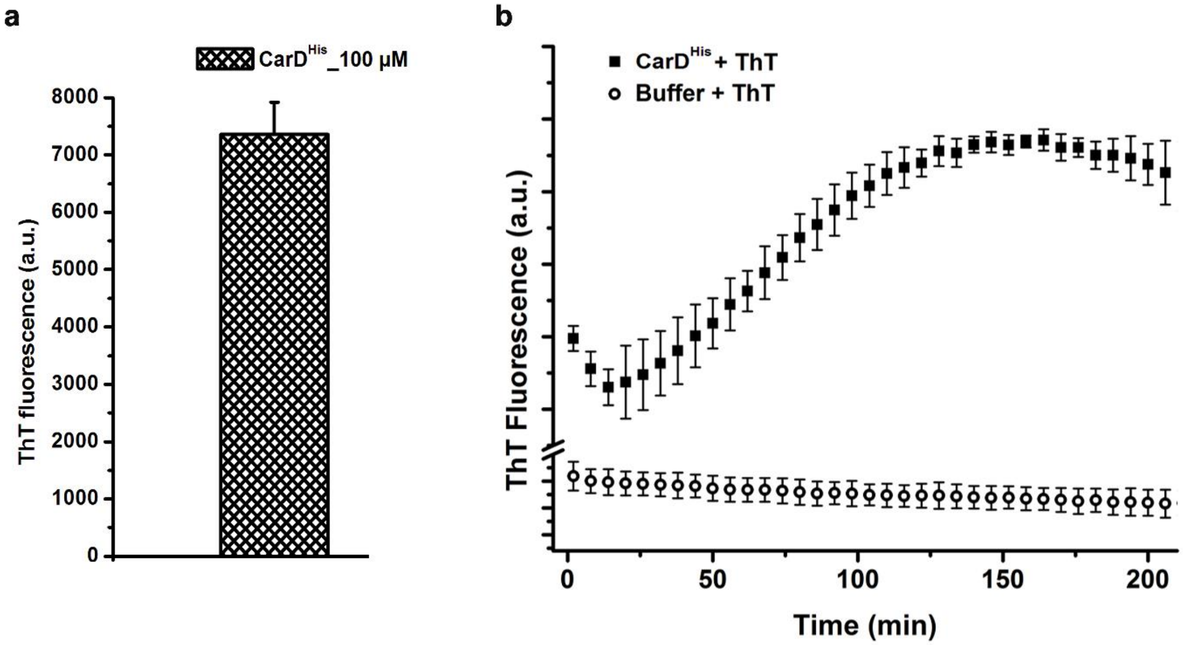

Supplementary Fig. 6: (a) CarD^{His} forms amyloid-like fibrils in solution. (a) Thioflavin T fluorescence assay: Staining of CarD^{His} (100 μM) with amyloid specific fluorescent dye, Thioflavin T (ThT) at 37 ºC after 15 min shows enhanced fluorescence intensity. The average fluorescence intensity and standard error bars have been calculated from three independent experiments. (b) The kinetics of amyloid fibril formation of CarD^{His} (100 μM) was monitored at 37 °C using ThT. The ThT fluorescence intensity was measured using excitation wavelength of 440 nm and emission wavelength of 482 nm.

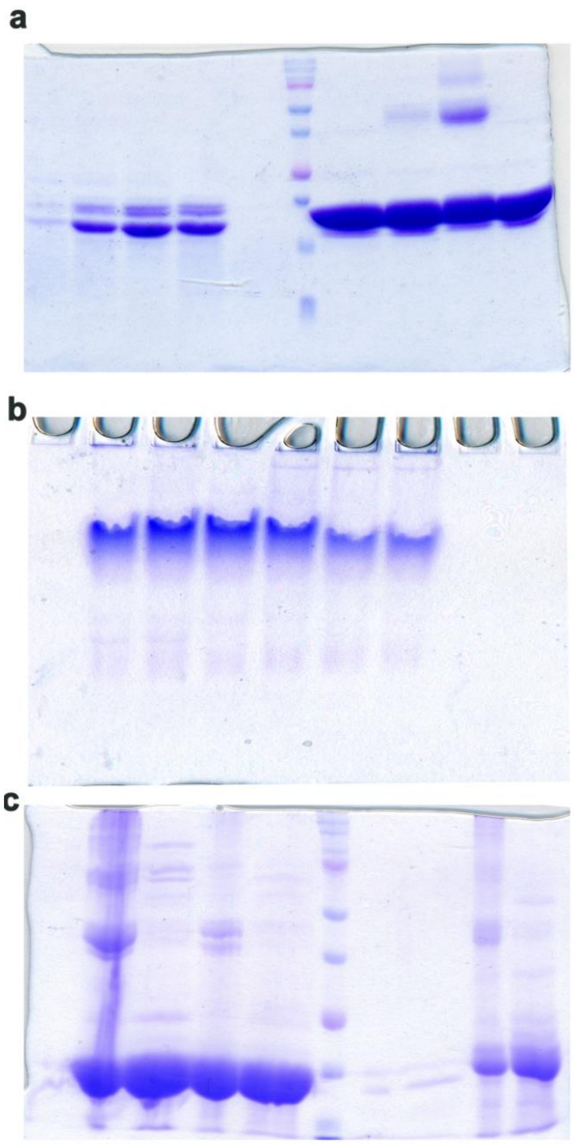

Supplementary Fig. 7: The un-cropped SDS-gels shown in panels a-c corresponds to the Fig. 5b-d, respectively in the main text.
